# *LRBA* is Essential for Allogeneic Responses in Bone Marrow Transplantation

**DOI:** 10.1038/srep36568

**Published:** 2016-11-08

**Authors:** Mi Young Park, Raki Sudan, Neetu Srivastava, Sudha Neelam, Christie Youngs, Jia-Wang Wang, Robert W. Engelman, William G. Kerr

**Affiliations:** 1 Department of Microbiology & Immunology, SUNY Upstate Medical University, Syracuse, NY, US; 2 Department of Internal Medicine, University of South Florida, Tampa, Florida, US; 3 Department of Pathology & Cell Biology, University of South Florida, Tampa, Florida, US; 4 Department of Pediatrics, University of South Florida, Tampa, Florida, US; 5 H. Lee Moffitt Comprehensive Cancer Center & Research Institute; University of South Florida, Tampa, FL; 6 Department of Biochemistry & Molecular Biology, SUNY Upstate Medical University, Syracuse, NY, US; 7 Department of Pediatrics, SUNY Upstate Medical University, Syracuse, NY, US; 8 Chemistry Department, Syracuse University, Syracuse, NY 13210 USA

## Abstract

The PH-BEACH-WD40 (PBW) protein family members play a role in coordinating receptor signaling and intracellular vesicle trafficking. *LPS-Responsive-Beige-like Anchor (LRBA*) is a PBW protein whose immune function remains elusive. Here we show that *LRBA*-null mice are viable, but exhibit compromised rejection of allogeneic, xenogeneic and missing self bone-marrow grafts. Further, we demonstrate that *LRBA*-null Natural Killer (NK) cells exhibit impaired signaling by the key NK activating receptors, NKp46 and NKG2D. However, induction of IFN-γ by cytokines remains intact, indicating *LRBA* selectively facilitates signals by receptors for ligands expressed on the surface of NK targets. Surprisingly, *LRBA* limits immunoregulatory cell numbers in tissues where GvHD is primed or initiated, and consistent with this *LRBA*-null mice also demonstrate resistance to lethal GvHD. These findings demonstrate that *LRBA* is redundant for host longevity while being essential for both host and donor-mediated immune responses and thus represents a unique and novel molecular target in transplant immunology.

Via a gene-trap approach we identified several novel LPS-responsive genes including the 7a65 gene-trap that harbored a *lacZ* gene-trap integration into a gene we subsequently cloned and designated *LPS-Responsive-Beige-like Anchor (LRBA*)^1^,^2^. The longest isoform, *LRBA-α* is a 2,856aa protein that contains a ConA-like/lectin domain (ConA), Armadillo/β-catenin-like repeats (ARM), PKA/C-binding motifs (AKAP), a conserved Domain of Unknown Function (DUF), Pleckstrin Homology (PH) domain, beige and Chediak-Higashi syndrome (BEACH) domain and a series of WD40 repeats at the COOH terminus (Figure S1A)^2^. In addition, we identified two other murine isoforms, *LRBA-β* and *-γ*, those possess unique amino acid stretches at their COOH termini, but also lack all five COOH terminal WD repeats (*LRBA-γ*) or the two most terminal repeats (*LRBA-*β)^2^. The latter is significant because omission of these WD40 repeats may alter the capacity of these shorter isoforms to partner with proteins recognized by these structures. Intriguingly, the *LRBA-α, -β*, and *-γ* isoforms are generated by alternative splicing and RT-PCR analysis indicates this regulation results in differential expression of the three isoforms across different tissues and even developmentally within a single cell lineage^2^. The human and murine *LRBA* proteins show 90% sequence homology^2^. *LRBA* is a member of highly conserved PH-BEACH-WD40 (PBW) protein family^3^,^4^. The PBW protein family harbors domains that have capacity to associate with cell membranes (PH, BEACH domains), glycoproteins (ConA-like lectin domain), but also protein-protein interaction domains (WD40 repeats, AKAP motif, ARM domain) and are predicted to serve a scaffolding function uniquely equipped to coordinate vesicle trafficking to sites of receptor signaling at the plasma membrane^2^,^4^,^5^.

*LRBA* is expressed ubiquitously in normal tissues, but its expression is increased in malignancy, particularly in breast cancer^6^,^7^. In immune cells *LRBA* is found in all membrane compartments associated with receptor signaling including the plasma membrane, golgi complex, clathrin-coated pits and trans-endocytic vesicles^2^. Based on its domain structure and subcellular localization, we proposed that *LRBA* coordinates signaling of immune receptors to promote effector function and thus plays a crucial role in immune regulation^2^. Consistent with this, several recently identified individuals with homozygous or compound heterozygous mutations in *LRBA* that result in *LRBA* protein deficiency suffer from immune dysregulation and manifest a spectrum of clinical complications including common variable immune deficiency (CVID), recurrent infections and autoimmunity that also includes inflammatory bowel disease (IBD)^8^,^9^,^10^,^11^. Further, recently in T-cells *LRBA* has been shown to regulate CTLA4 turnover hence, modulating CTLA4 mediated immune signaling^12^. These observations indeed thus support a crucial role of *LRBA* in immune regulation. However, our understanding of the effector functions regulated by *LRBA* and how *LRBA* regulates these immune cell effector functions is still very limited and remains to be studied.

Here, we report the first *LRBA*-null mouse model. We report that *LRBA*-null mice show normal life span but exhibit compromised rejection of allogeneic, xenogeneic and missing self bone-marrow (BM) grafts as well resistance to acute graft vs host disease (GvHD). NK cell analysis revealed that NK cells from these mice show defective NK cell activating receptor signaling. Further, *LRBA* regulates MDSC and Treg numbers in tissues where GvHD is primed. Thus our findings demonstrate a pivotal role of *LRBA* in NK effector functions and transplant immunology.

## Results

### *LRBA* Deficiency Compromises Allogeneic, Minor Histocompatibility Antigen (miHAg) Mismatched, Missing Self and Xenogeneic BM Graft Rejection

In order to assess a possible role for *LRBA* in cellular immunity, we generated *LRBA*-null mice (Null) by using embryonic stem (ES) cells where the *LRBA* gene is inactivated via gene-trap integration into the intron between exons 2 and 3 (Figure S1B). *LRBA* is abundantly expressed in kidney and brain^2^ and thus RNA from these tissues was used for Northern blot analysis to confirm that *LRBA*-null mice lack expression of *LRBA* mRNA (Figure S1C). Homozygous *LRBA*-null mice on a 129 Sv background had a normal and healthful lifespan that does not differ significantly from 129 Sv wild-type mice (WT) and heterozygous littermates (*LRBA*^+/−^) (Figure S1D). Consistent with their normal lifespan, full necropsy and careful histopathology analysis of all major organs performed on multiple *LRBA*-null mice did not reveal any significant abnormalities or pathologies (Figure S2). This included the gastrointestinal tract where IBD of various clinical types was reported in all humans homozygous for inactivating *LRBA* mutations^8^,^9^,^10^,^11^.

**Figure 1:**
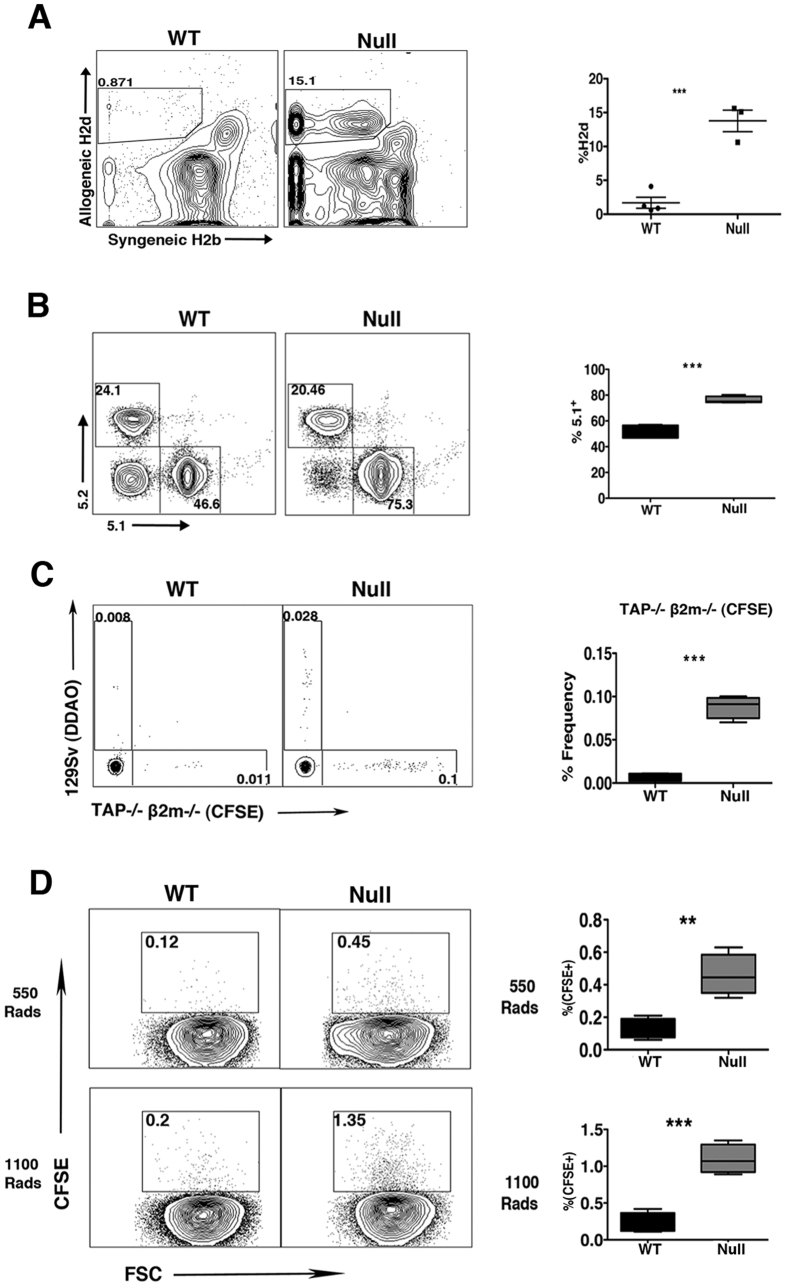
*LRBA*-null Hosts show Compromised Allogeneic, miHAg Mismatched, Missing Self and Xenogeneic BM Graft Rejection. **(A)** Equal number of Balb/c (H2^d^) and syngeneic 129Sv (H2^b^) BM cells were mixed and injected into lethally irradiated WT and *LRBA*-null (Null) mice. Contour plot and scatter plot indicating presence of circulating allogeneic donor cells 7 day post BM transplantation are shown. **(B)** Lethally irradiated WT and Null mice received equal number of C57BL6 (CD45.1) miHAg mismatched and syngeneic 129Sv (CD45.2) BM cells. Thirty days post-BM transplantation splenocytes were analysed for presence of Ly5.1 and Ly5.2 cells by flow cytometry. **(C)** Equal number of TAP2^−/−^β2m^−/−^ (missing self) BM cells stained with CFSE and 129Sv BM cells stained with DDAO were mixed and injected into lethally irradiated WT or Null hosts. Mice were bled and analysed for presence of TAP2^−/−^β2m^**−/−**^ BM cells after 18 hrs and data is presented as contour and Box-and-whisker plot. **(D)** Sub-lethally (550cGy) or lethally irradiated WT or Null hosts received CFSE labeled human BM cells. After 18 hrs mice were bled and analysed for presence of CFSE^+^ human cells by flow cytometry. Results shown in A are representative of 3 independent experiments with n = 3–4 mice per group. Results shown in B, C and D are representative of two independent experiments with n = 3–4 animals per group. *p < 0.05, **p < 0.01,***p < 0.001, ****p < 0.0001.

To provide an initial assessment of *LRBA*’s importance in cellular immunity, we compared the ability of *LRBA*-null mice on a 129Sv (H2b, Ly5.2+) background to acutely reject BM grafts from MHC-I mismatched (H2d) BALB/C donors. To accomplish this, allogeneic BALB/C BM cells were mixed with syngeneic 129Sv BM cells at 1:1 ratio and injected into lethally-irradiated *LRBA*-null or WT 129Sv hosts. 7 days later mice were bled and the presence of allogeneic donor cells was analyzed by flow cytometry (Fig. 1A). We observed a significantly greater frequency of allogeneic donor cells in *LRBA*-null hosts relative to WT hosts (Fig. 1A) suggesting that acute rejection of MHC-I unmatched BM is compromised in *LRBA*-null hosts. We then analyzed BM graft rejection in the context of extensive miHAg mismatch between donor and host. B6.Ly5.1 (H2b, Ly5.1^+^) BM cells were mixed with syngeneic BM cells at 1:1 ratio and injected into *LRBA*-null or WT hosts (Fig. 1B). Thirty days post-transplant splenocytes from WT and *LRBA*-null mice were analyzed for the presence of donor cells. We observed significantly increased frequency of miHAg mismatched (B6.Ly5.1) donor cells in *LRBA*-null hosts as compared to WT (Fig. 1B). These findings implicate *LRBA* as being essential for efficient rejection of MHC-I mismatched donor BM stem/progenitors, a cellular immune function primarily mediated by NK cells. However, diminished rejection of miHAg mismatched grafts indicates the residual T-cell immune barrier in lethally irradiated hosts may also be impaired by *LRBA*-deficiency, and thus *LRBA* could also contribute to cytotoxic T-cell function. Although NK cells can also contribute to rejection of miHAg mismatched BM grafts, due to allelic variation in mitochondrial antigens^13^. Thus, enhanced miHAg engraftment may also reflect NK dysfunction in *LRBA*-null hosts.

As shown by Karre and colleagues for tumor cells^14^ and subsequently by Bix *et al*. for β2m^−/−^ BM grafts^15^, there is a unique immune role for NK cells in the killing and rejection of *missing self* tumor cells and BM grafts that lack surface expression of MHC-I. To further confirm that *LRBA* is required for NK function *in vivo*, we challenged *LRBA*-null and WT hosts with MHC-I deficient donor BM cells from TAP2^−/−^β2m^−/−^ hosts. As with allogeneic donor cells, we observed significantly enhanced acute engraftment of CFSE-labeled missing self TAP2^−/−^β2m^−/−^ cells (Fig. 1C). As NK cells play a unique role in rejection of missing self grafts these findings unequivocally implicate *LRBA* in the *in-vivo* cytotoxic function of NK cells. Improved engraftment of missing self BM cells in *LRBA*-null hosts suggested that a xenogeneic graft might also be rejected to a lesser degree by *LRBA*-null hosts. To test this possibility we challenged *LRBA*-null and WT hosts with human BM cells and assessed acute engraftment the next day. As with missing self grafts, we also observed significantly improved human cell engraftment in *LRBA*-null hosts as compared to WT, when hosts were conditioned prior to transplant with myeloablative radiation doses (Fig. 1D).

### *LRBA* is Required for Efficient Signaling by Key NK Activating Receptors

**Figure 2:**
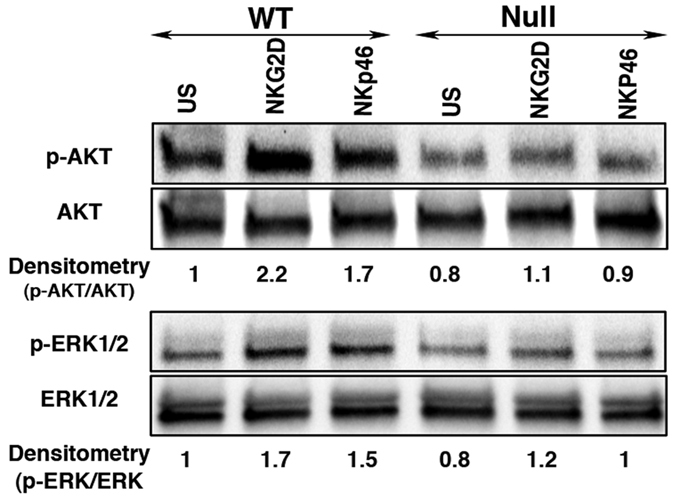
*LRBA*-null NK cells show compromised AKT and ERK1/2 MAPK phosphorylation. Immunoblot analysis of AKT and ERK1/2 phosphorylation from IL-2 expanded NK cells after stimulation with plate bound NKG2D and NKp46 antibodies for 15 minutes. Numbers below the blot indicate densitometry ratio. Experiment was repeated twice and representative blots from one experiment are shown.

**Figure 3:**
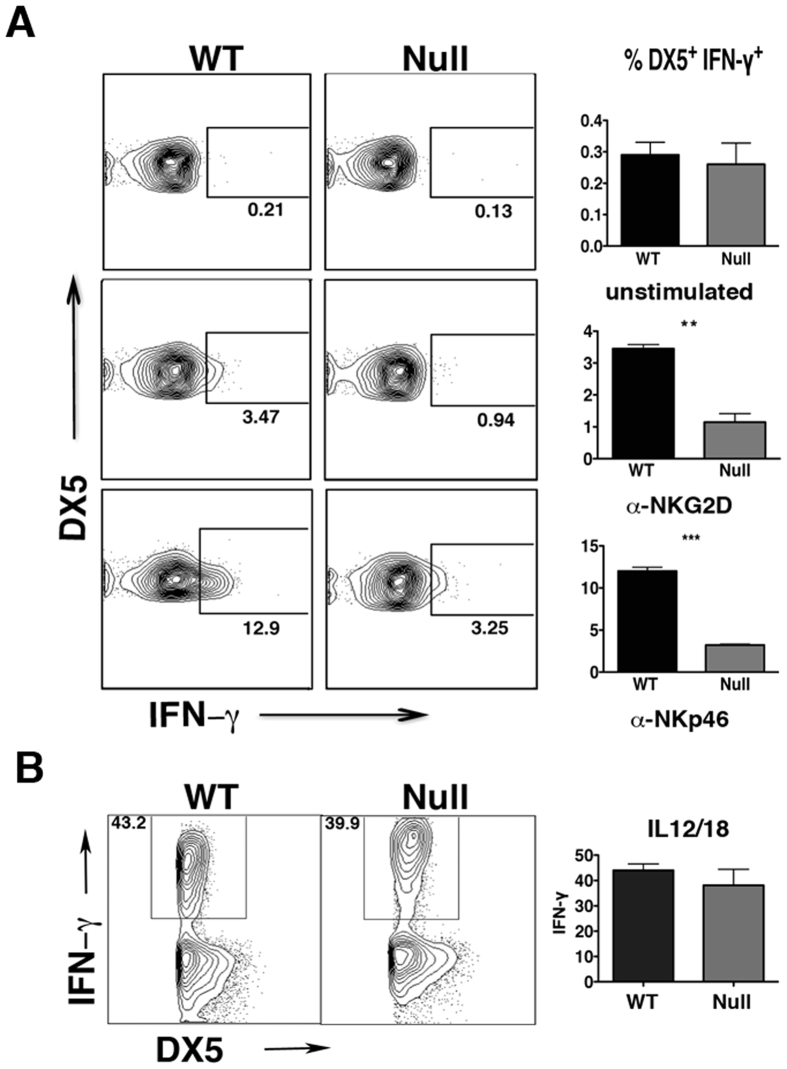
*LRBA* is required for NK activating receptors to induce IFN-γ. (**A**) Splenocytes from WT and Null mice, injected with polyI:C were stimulated with 50μg of plate-bound α-NKp46 or α-NKG2D antibodies. The induction of IFN-γ was analyzed by intracellular cytokine staining. Plots show the frequency of IFN-γ + NK cells after gating on CD49b(DX5)^+^ TcRβ^−^ cells. **(B)** Splenocytes from unmanipulated WT and LRBA-null mice were stimulated overnight using IL12 and IL18. The production of IFN-γ was then analyzed by flow cytometry. Statistical analysis of LRBA-null and WT NK cell production of IFN-γ showed no significant difference (p = 0.4). Results are representative of three independent experiments (A-B). **p < 0.05, ***p < 0.0001.

To provide mechanistic insights into the NK cell functional deficit identified, we initially assessed NK cell numbers and terminal maturation by CD11b and CD27 staining^16^. This analysis revealed that NK cells are present at normal numbers in *LRBA*-null mice and exhibit normal maturation (Figure S3A,B). As NKG2D has been implicated in NK mediated rejection of allogeneic BM grafts^17^, while NKp46 has been shown to be necessary for NK responses to influenza and certain tumor types^18^,^19^, we then examined the expression and signaling capacity of these two key NK activating receptors. Flow cytometric analysis showed that both NKp46 and NKG2D are expressed normally on peripheral NK cells in *LRBA*-null mice (Figure S3C). Also, *LRBA*-null mice had unchanged T-cell and B-cell frequency compared to WT mice (Figure S4A–D). We then examined the signaling capacity of these two NK activating receptors by assessing their ability to induce phosphorylation of two key signaling molecules, AKT and ERK1/2 MAPK^20^,^21^, and IFN-γ expression. When MACS purified, IL-2 expanded NK cells from WT and *LRBA*-null mice were stimulated with plate bound agonistic anti-NKG2D and anti-NKp46 antibodies, we observed impaired ERK1/2 and AKT phosphorylation in *LRBA*-null NK cells compared to WT suggesting signaling defects in *LRBA*-null NK cells (Fig. 2). We also observed that the engagement of either NKp46 or NKG2D was found to induce IFN-γ expression in a significantly lower proportion of *LRBA*-null splenic NK cells compared to WT (Fig. 3A). IFN-γ, is a major NK effector cytokine, and ability to induce IFN-γ is widely utilized to test the functional competency of NK cells^22^,^23^,^24^. Inability to induce AKT and ERK1/2 MAPK phosphorylation, and IFN-γ suggests that signaling of both NKp46 and NKG2D is impaired in the *LRBA*-null NK cells. In order to determine whether *LRBA*-null NK cells are broadly disabled for induction of IFN-γ we also assessed whether co-stimulation with IL-12 and IL-18 is able to induce IFN-γ in these cells. Multiple independent experiments showed that IFN-γ induction in *LRBA*-null NK cells was intact following co-stimulation with this cytokine pair (Fig. 3B) that can potently activate NK effector functions independent of activating receptor engagement^25^. Thus, *LRBA*-null NK cells are disabled for induction of effector responses triggered by receptors like NKG2D and NKp46 that recognize surface-elaborated ligands on target cells and not soluble factors that promote their differentiation and effector function.

### *LRBA* is Required for MIP-1α Production and Cytolytic Function of NK Cells

**Figure 4:**
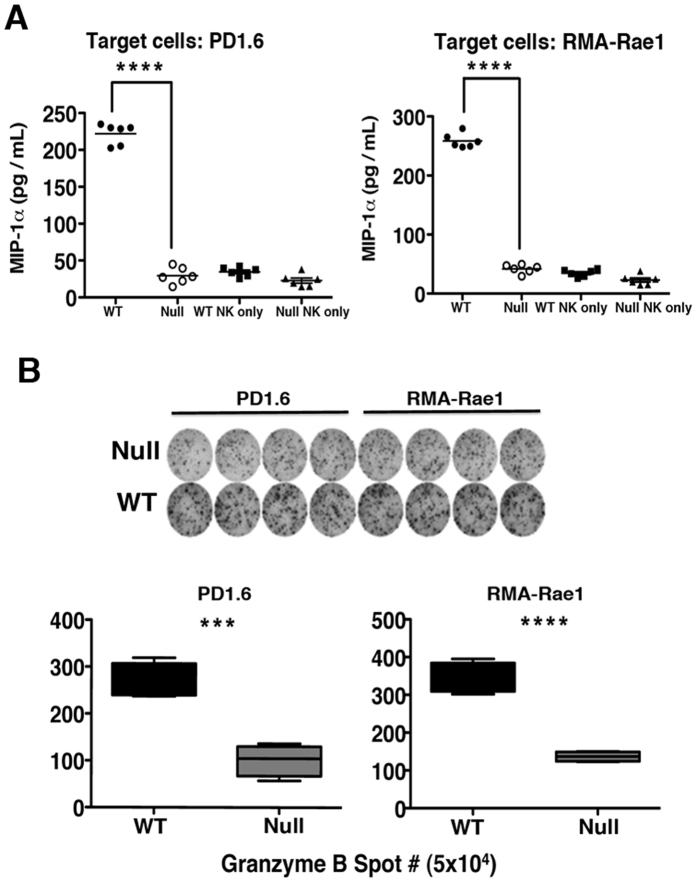
*LRBA* plays a pivotal role in induction of MIP-1α and cytolytic function. **(A)** The NK cells were isolated from WT or *LRBA*-null spleens by MACS and co-incubated with PD1.6 or RMA-Rae1 tumor cells in a 96-well multi-test plate at 37 °C, 5% CO_2_ for 20 hr. Cell-free supernatant was harvested and MIP-1α production measured by ELISA assay. **(B)** The multi-test plate was used to determine the number of Granzyme B secreting NK cells by ELISpot assay. The data in (A) and (B) are representative of three independent experiments.***p < 0.001,****p < 0.0001.

We further examined whether defective NKp46 and NKG2D signaling by *LRBA*-null NK cells also applied to their ligands when expressed on the surface of target cells - a more physiologically relevant means of receptor engagement than antibody cross linking. We tested this by incubating *LRBA*-null and WT NK cells with PD1.6 and RMA-Rae1 targets cells that express NKp46^19^ and NKG2D ligands, respectively, and then analyzed production of macrophage activating factor MIP1α (Fig. 4A) and granzyme B secretion (Fig. 4B). The latter assay provides a measure of the capacity of NKp46 and NKG2D to trigger cytolytic function. We observed significantly diminished MIP1α production and cytolytic responses toward PD1.6 and RMA-Rae1 target cells, confirming that both NKp46 and NKG2D signaling are defective in *LRBA*-null NK cells, and importantly with bonafide cellular targets expressing their respective ligands. The defective NKp46 and NKG2D signaling that we observe using three independent measures indicates *LRBA* facilitates signaling downstream of these key NK activating receptors.

### *LRBA* Deficiency Promotes Resistance to GvHD

**Figure 5:**
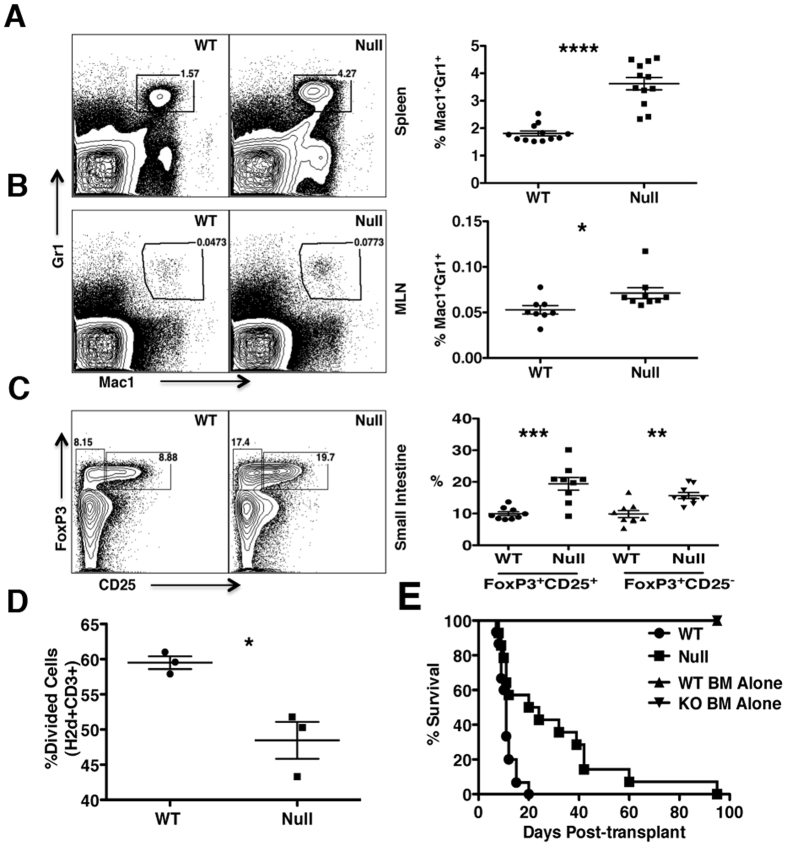
*LRBA* deficiency enhances resistance to GvHD. **(A)** Representative contour plots (left) showing Mac1^+^ Gr1^+^ MDSC after gating for viable cells from splenocytes of WT and *LRBA*-Null mice and scatter plot (right) showing the frequency of Mac1^+^ Gr1^+^ MDSC (pooled data from 3 experiments is shown, with total N = 12, ****p < 0.0001). **(B)** Contour plot (left) and scatter plot (right) showing frequency of Mac1^+^ Gr1^+^ MDSC in the mesenteric lymph node (MLN) of WT and Null mice (pooled data from 3 experiments is shown, N = 8–9, *p < 0.05). **(C)** Flow cytometry plots (left) and scatter plot (right) indicating frequency of FoxP3^+^ CD25^+^ (nTreg) and FoxP3^+^ CD25^−^ (iTreg) cells gated on live CD3^+^ CD4^+^ small intestinal lamina propria lymphocytes from WT and Null mice (pooled data from 3 experiments is shown, N = 9, ***p < 0.001, **p < 0.01). **(D)** Percentage of divided (CFSE diluted) BALB/c responder (CD3+H2d+) cells co-cultured with irradiated LRBA null and WT stimulators in one way MLR assay is shown. Experiment is representative of two independent experiments (*p < 0.05). **(E)** Lethally irradiated 129Sv WT (n = 15) and *LRBA*-null hosts (n = 14) received 1 × 10^7^ T-cell depleted BM cells plus 2 × 10^6^ purified splenic T-cells from BALB/c donors whereas WT and Null controls (n = 5) received T-cell depleted BM cells only. Mice were monitored for survival and data is presented as a step-function (**p < 0.01, log-rank test).

Host vs. graft (HvG) rejection is only one of two immunological hurdles that must be overcome in order for a host to survive a myeloablative allogeneic BMT procedure. The graft vs. host immune response, that culminates in GvHD, constitutes the second and most formidable barrier to host survival^26^. As documented above *LRBA*-null hosts have a compromised HvG barrier and thus we considered the possibility that they might also prime a poor GvH response that lead to lethal GvHD. Consistent with this possibility, we find that two major immunoregulatory cells, MDSC and Treg cells, that are known to limit GvHD^27^,^28^,^29^,^30^,^31^, are increased in *LRBA*-null hosts at immune locations where GvHD is initiated. We find that the frequency of MDSC (Mac1+ Gr1+) is significantly increased in both the spleen (Fig. 5A) and mesenteric lymph node (MLN) (Fig. 5B) of *LRBA*-null mice, while Treg cells, including both native (nTreg) and induced Treg (iTreg) populations are significantly increased in the small intestine of *LRBA*-null hosts (Fig. 5C). Because of the increased numbers of both immunoregulatory cell types, we then assessed the capacity of *LRBA*-null splenocytes to prime donor allogeneic T-cell responses in a one-way mixed lymphocyte reaction (MLR) assay. Consistent with the increased numbers of MDSC in *LRBA*-null spleen we observed that *LRBA*-null stimulators prime allogeneic T-cell proliferation poorly as compared to WT stimulators (Fig. 5D). The one-way MLR is generally considered an *in-vitro* surrogate for GvHD; however, it may only be relevant to donor T-cell responses with cells from tissues used in the assay. Thus, we performed a lethal GvHD challenge in *LRBA*-null hosts to determine if suppression of donor T-cell responses by *LRBA* host tissues occurs *in vivo* as measured by host survival. Using a standard lethal acute GvHD BMT model^32^, we find that *LRBA*-null hosts exhibit enhanced survival in this setting (Fig. 5E). Thus, *LRBA* deficiency also abrogates donor allogeneic immune responses that culminate in lethal acute GvHD during MHC-I unmatched BMT.

## Discussion

Recent reports identifying immune dysfunction in *LRBA*-deficient patients suggest a critical role of *LRBA* in immune homeostasis. The induction of *LRBA* expression by mitogen and its ability to traffic to the plasma membrane or sites of receptor-mediated endocytosis suggested *LRBA* might have a role in receptor signaling by immune effector cells^2^. However, our understanding of the effector functions regulated by *LRBA* and how *LRBA* regulates these immune cell effector functions is very limited and remains to be studied. Here, we studied the role of *LRBA* in regulating NK effector functions and BM transplantation. We show that *LRBA* is pivotal for NK cell mediated rejection of allogeneic, missing self and xenogeneic hematopoietic grafts and may also be required for T-cell responses to miHAg mismatched grafts. However, NK cells can also distinguish and reject miHAg mismatched cells based on variation in mitochondrial DNA content via MyD88 signaling^13^. Thus, it is possible that *LRBA* could also facilitate MyD88-mediated NK rejection of miHAg mismatched BM grafts. In addition, *LRBA* is also required for homeostatic control of MDSC and Treg cells and consistent with this *LRBA*-null hosts show significant resistance to acute GvHD following MHC-I unmatched BMT. Inability to reject allogeneic BM grafts and resistance to acute GvHD observed in *LRBA*-null mice suggests the possibility that the recently identified *LRBA*-deficient patients are strong candidates to benefit from allogeneic BMT^8^,^9^,^10^,^11^.

Our analysis indicates *LRBA* selectively facilitates receptor signals from major NK activating receptors like NKp46 and NKG2D that interact with ligands expressed on the surface of virally infected, stressed or malignant targets. However, *LRBA* does not appear to be required for signaling by soluble inducers of NK effector function like IL12 and IL18. This distinction may provide insights into *LRBA*’s role in promoting NK effector functions. PBW proteins possess domains that can associate with inositol phospholipid species^33^,^34^, vesicular membranes^2^ and plasma membrane sites involved in receptor signaling^2^. Like other members of the PBW protein family, *LRBA* possesses domains that could enable its recruitment to the plasma membrane at sites near to the cellular receptors, and thus can coordinate recruitment of vesicles containing effector cytokines or cytolytic proteins to enable efficient secretion and targeting of these cargoes toward their targets. Cytoplasmic granules within CTL and NK cells release Granzyme B that induce apoptosis within virus-infected cells or tumor cells. As predicted, *LRBA*-null NK cells stimulated with either PD1.6 or RMA-Rae1 exhibited greatly impaired secretion of both MIP-1α and Granzyme B and also exhibited impaired phosphorylation of AKT and ERK1/2 MAPK as compared with WT control suggesting that *LRBA* can also facilitate the PI3K-RacI-PAK-MEK-ERK pathway that promotes trafficking of lytic granules in NK cells toward targets cells^20^,^35^. *LRBA* thus may be coordinating receptor signaling events with effector functions in NK cells by regulating efficient trafficking of cytolytic granules or cytokines downstream of activating receptors such as NKp46 and NKG2D. In this regard it is intriguing that *Neurobeachin*, the PBW paralog with the highest degree of homology to LRBA, is required for synapse function in neuronal cells^33^,^34^,^36^. Perhaps LRBA has a comparable role in the synapse that NK cells form with their targets. This could account for the fact that LRBA deficiency does not impair induction of NK effector function by soluble activators like IL12 and 18, but is critical for responses to ligands presented on the surface of target cells. Also, NK cell IFN-γ is known to have direct and indirect effects on other immune cells like T-cells and DCs^37^. IFN-γ can induce maturation of DC leading to IL12 production and increased co-stimulation. DC maturation in turns promotes enhanced T-cell responses^37^. Low IFN-γ production by Null NK cells thus means there could be reduced or compromised DC activation and priming of T cells. That might also explain defective priming of allogeneic T-cells by *LRBA*-null cells in our MLR and GvHD assays. Further, defective NKG2D and NKp46 signaling observed in *LRBA*-null NK cells caution us that *LRBA*-deficient patients may be highly susceptible to tumors and viral infections as these two receptors are crucial for anti-tumor and anti viral immune responses^17^,^18^,^19^. Indeed, in a recent paper Gamez-Diaz *et al*., reported that in a cohort of 22 *LRBA*-deficient patients viral infections were the most frequent infections and 9/22 patients (41%) suffered from them. Common viral infections were cytomegalovirus, adenovirus, norovirus, and herpes virus. Thus clearly suggesting NK cell defects^11^.

Contrary to its apparent positive signaling role in promoting NK effector function, *LRBA* appears to play a role in signals that limit the numbers of MDSC and Treg cells. Intriguingly, the latter role for *LRBA* is quite tissue specific as n and iTreg increases were only observed in the small intestine of *LRBA*-null hosts. Interestingly, GvHD is thought to be initiated in the gut where myeloablative strategies such as total body irradiation compromise the integrity of the gut epithelium leading to commensal encroachment and consequently an inflammatory milieu that supports efficient priming of donor T-cells that mediate lethal GvHD^38^. Perhaps increased Treg cell numbers there limit the initiation of GvHD in the *LRBA*-deficient host with increased MDSC numbers further dampening the spread of GvHD to other tissues by donor T-cells that escape control by Treg cells in the small intestine.

The improved durable engraftment of MHC class I- and miHAg-mismatched BM grafts with reduced incidence and severity of GvHD that we observe in *LRBA*-deficient mice suggests the possibility that the recently identified *LRBA*-deficient patients are strong candidates to benefit from allogeneic BMT using related HLA haploidentical or HLA-matched donors^8^,^9^,^10^,^11^. In this regard, the longevity of *LRBA*-null mice and the absence of any detectable pathology in non-hematopoietic organs and tissues indicate hematolymphoid reconstitution from allogeneic *LRBA*-sufficient donors would enable long-term survival in these transplanted patients, as *LRBA*-deficiency does not have a significant effect on the function and longevity of non-hematopoietic tissues and organs. Indeed recently, a small subset of *LRBA*-deficient patients were treated with HSCT. Out of 6 patients treated so far 4 patients survived and 2 of the patients died^11^,^39^,^40^,^41^. One patient died from respiratory failure and the 2^nd^ patient died of thrombotic thrombocytopenic purpura^11^. However, despite that only a handful of patients being treated the success rate of HSCT is remarkable that clearly underlines the importance of our study. Findings from our study thus clearly support that *LRBA*-deficient patients are good candidate for receiving a HSCT. However, caution should be applied before extrapolating findings from mouse study to humans as *LRBA*-deficient patients are progeny of consanguine matings, it’s possible that they are homozygous for many other alleles that could, in concert with *LRBA* deficiency, cause the observed immune phenotypes in these individuals. Indeed, a high degree of variation in the occurrence and severity of spectrum of disease phenotypes in *LRBA*-deficient patients strongly suggests that *LRBA*-deficiency is modified by alleles at other loci to promote disease in these patients. However, that is not the case with the *LRBA*-null mice on a defined genetic background. Further, in case of human subjects results could vary from individual to individual, as there could be many factors that can effect the outcome of a HSCT that includes:- the disease progression stage (early vs late) at which HSCT is performed, the conditioning regiment used for lymphodepletion, the donor source, the post-transplant recovery, post-transplantation viral infections (patient 1) and sepsis and GvHD. Thus, risk of allogeneic HSCT should be weighed against the clinical presentation in each individual case. Further, our finding that xenogeneic human BM cells are rejected less efficiently in *LRBA*-null hosts may have practical implications for the development of novel immunodeficient mouse models of human immunity and hematopoiesis where *LRBA* mutation could be combined to other immune deficient mutations. Collectively, our study suggests that *LRBA* represents truly unique molecular target in transplant immunology, as it plays a pivotal role in both HvG and GvH immune reactions while not being required for host viability.

## Experimental Procedures

### Mice

B6.Ly5.1 (H2^b^ Ly5.1^+^) or BALB/c (H2^d^) were purchased from Jackson and 129Sv (H2^b^ Ly5.2^+^) or TAP2^−/−^β_2_m^−/−^ mice were purchased from Taconic. All mice were used at the age of 8–12 weeks. *LRBA*^−/−^ mice were derived via blastocyst injection of embryonic stem cells harboring a gene-trap retrovirus^42^ whose integration in the *LRBA* gene inactivates its expression by introduction of an artificial exon that terminates endogenous *LRBA* transcription and splicing to downstream exons. Southern blotting was used to identify murine progeny with significant transmission of the *LRBA* gene-trap integration and these chimeric mice were then crossed to 129Sv partners to obtain mice that have germline transmission of the *LRBA* mutant allele on a pure 129Sv genetic background. These *LRBA*^+/−^ progeny were then intercrossed to obtain *LRBA*-null homozygous mice with confirmation of the null homozygous genotype by a multiplex PCR. Northern blotting of kidneys and brains, two tissues that have abundant *LRBA* expression, confirmed that *LRBA*-null mice lack detectable mRNA expression from the *LRBA* locus. All animal experiments were approved by Institutional Animal Care and Use Committee (IACUC) of SUNY Upstate Medical University and performed in accordance with approved guidelines.

### Flow cytometry analysis

Antibodies were purchased from BD Biosciences (San Jose, CA) or eBioscience (San Diego, CA). Samples were Fc blocked with anti-CD16/CD32 and then stained with the indicated antibodies and acquired on LSRII or Fortessa. Various antibodies used for staining are: CD49b-PE (DX5), CD3-PerCPCy5.5 (145-2C11), CD335-FITC (NKp46, 29A1.4), CD314-PE Cy-7 (NKG2D, CX5), H2^d^-FITC (34-2-12) and H2^b^-PE (AF6-88.5) or H2^b^-PECy7, CD45.1-PE (A20) and CD45.2-FITC (104). For MDSC analysis CD11b-APC-Cy7(M1/70) and Gr1(RB6-8C5)-PE antibodies were used. For Treg cell analysis small intestine lamina propria T cells were isolated as described previously^43^ and stained with CD3 PECy7 (145-2C11), CD4 FITC (RM4-5), CD25 APC (PC61.5) and Foxp3 PE (FJK-16s) antibodies.

### IFN-γ assay

For licensing assays, mice were injected i.p. with 70 μg of Poly IC on day –1. Spleens were harvested next day, RBC lysed and resuspended in RPMI 1640 supplemented with 10% FBS, 1% penicillin-streptomycin, 1% L-glutamine, 1% sodium pyruvate, and 1% nonessential amino acids. 4–6 million splenocytes were incubated with plate bound anti-NKG2D (A10) or anti-NKp46 (29A1.4) in 6-well plates for 5–6 h at 37 °C in the presence of GolgiPlug. The cells were harvested, and stained for CD49b-PE(DX5) and TCR-FITC (H57-597) and then permeabilized and fixed using the eBioscience Fixation/Permeabilization kit and stained with anti-IFN-γ-APC (XMG1.2). To stimulate NK cells using IL12/18, 5–6 million splenocytes were incubated in 6 well plates overnight in presence of 20ng/well IL12 and 100ng/well IL18. The next morning cells were incubated for 3–4 hr at 37 °C in the presence of GolgiPlug and harvested for flow analysis.

### ELISPOT assays

*LRBA*-null or WT NK cells were added to a 96-well granzyme B ELISpot plate at a concentration of 5 × 10^4^ cells/well. 1 × 10^4^ PD1.6 or RMA-Rae1^+^ cells were then added to the well as a stimulus for NK cells and microplate was incubated for 20 hr at 37□C in a CO_2_ incubator. Cells were removed and the supernatant was harvested to perform MIP-1α ELISA assay. ELISPOT assay was performed according to the manufacturer’s protocol (EL1865; R&D Systems). The number of spots corresponding to Granzyme B-secreting cells was determined using an automatic CTL ELISPOT Reader (Shaker Heights, OH). The number of background spots in wells without target cells was subtracted from the wells containing target cells and NK cells.

### Assessment of MIP-1α levels by ELISA assay

MIP-1α ELISA kit (MMA00) was purchased from R&D Systems Inc. (Minneapolis, MN) and MIP-1α ELISA was performed according to the manufacturer’s protocol.

### Dye labeling and flow cytometry based detection of donor cells *in vivo*

For dye labeling BM cells were labelled with CFSE or DDAO-SE (Invitrogen, Carlsbad, CA) at a concentration of 5μM in PBS at 37 °C for 15 minutes. For missing self acute engraftment, 1 × 10^7^ TAP2^−/−^β2m^−/−^ BM cells stained with CFSE and 1 × 10^7^ 129Sv (H2^b+^) BM cells stained with DDAO were mixed at 1:1 ratio and injected intravenously into lethally irradiated (1100Rad, split dose 600 + 500) WT or *LRBA*-null hosts. After 18hr PBMC from these mice were analyzed by flow cytometry for CFSE^+^ and DDAO^+^ cells. For 7 days acute engraftment, 2 × 10^6^ Balb/c (H2^d^) BM cells and 2 × 10^6^ syngeneic 129Sv (H2^b^) BM cells (4 × 10^6^ BM cells total/host) were infused intravenously via retro-orbital injection into lethally irradiated WT or *LRBA*-null hosts. To assess engraftment, peripheral blood mononuclear cells were harvested 7 days after BMT and stained for H2d and H2b and analyzed by flow cytometry. For minor histocompatibility antigen (miHAg) mismatched BM graft rejection splenocyte from recipients were harvested one month following BMT and analyzed. For xenogeneic transplantation human BM from healthy donors was purchased from Lonza (Allendale, NJ). CFSE labeled 5 × 10^6^ human BM cells were intravenously injected into WT or *LRBA*-null hosts that received either 550 or 1100Rads. After 18 hours the recipients were bled and analyzed by flow cytometry.

### Immunoblot Analysis

For Immunoblot analysis MACS purified NK cells were expanded in presence of recombinant human IL2 for 7 days. Cells were IL2 starved overnight and stimulated with plate bound NKG2D (A10) and NKp46 antibodies for 15 minutes. After 15 minutes cells were lysed in cell lysis buffer (Cell Signaling Technology) and equal amount of protein was run on SDS-PAGE. Proteins were transferred to nitrocellulose membrane and probed with p-ERK1/2, ERK1/2, p-AKT (Ser473) and AKT antibodies, all from Cell Signaling Technology except AKT which was from Santa Cruz. Immuno-reactive bands were visualized using ECL reagent (Thermo Scientific).

### GvHD

129Sv WT and *LRBA*-null mice were lethally irradiated followed by infusion of 10^7^ CD3-depleted BALB/c bone marrow cells plus 2 × 10^6^ splenic MACS purified T-cells via tail vein injection. Mice were monitored for survival and difference in animal survival were analyzed using a Kaplan-Meier log-rank test.

### Mixed Lymphocyte Reaction (MLR)

RBC lysed splenocytes from LRBA-null and WT mice were irradiated at 2000 rad (Stimulators) and co-cultured with CFSE labeled RBC lysed BALB/c splenocytes (responders) at a ratio of 2:1 in a one way MLR assay^28^,^44^. On day 4^th^ proliferation of responder T cells (H2d+CD3+) was assessed by flow cytometric analysis of CFSE dilution^44^.

### Statistical analysis

Data were analyzed for statistical significance using Prism (GraphPad, San Diego, CA). Students t-test was used to calculate the significance between groups. Differences in animal survival (Kaplan Meier curves) were analysed by log-rank test. Results were considered significant if p value is ≤0.05.

## Additional Information

**How to cite this article**: Park, M. Y. *et al. LRBA* is Essential for Allogeneic Responses in Bone Marrow Transplantation. *Sci. Rep.* **6**, 36568; doi: 10.1038/srep36568 (2016).

**Publisher’s note:** Springer Nature remains neutral with regard to jurisdictional claims in published maps and institutional affiliations.

## Supplementary Material

Supplementary Information
