# Supplementary material for: LRBA is Essential for Allogeneic Responses in Bone Marrow Transplantation
Source: Sci Rep. 2016 Nov 8;6:36568. doi: 10.1038/srep36568 (PMC5099895; doi:10.1038/srep36568)
Supplement: Supplementary Information [file srep36568-s1.pdf]

LRBA is Essential for Allogeneic Responses in Bone Marrow Transplantation

Mi Young Park,[1#] Raki Sudan,[1#] Neetu Srivastava,[1] Sudha Neelam,[1] Christie Youngs,[1] Jia-Wang Wang,[4] Robert W. Engelman,[5,6,7] and William G. Kerr[1,2,3,8*]

# contributed equally.

* To whom correspondence should be addressed: kerrw@upstate.edu

SUPPLEMENTAL FIGURES AND FIGURE LEGENDS

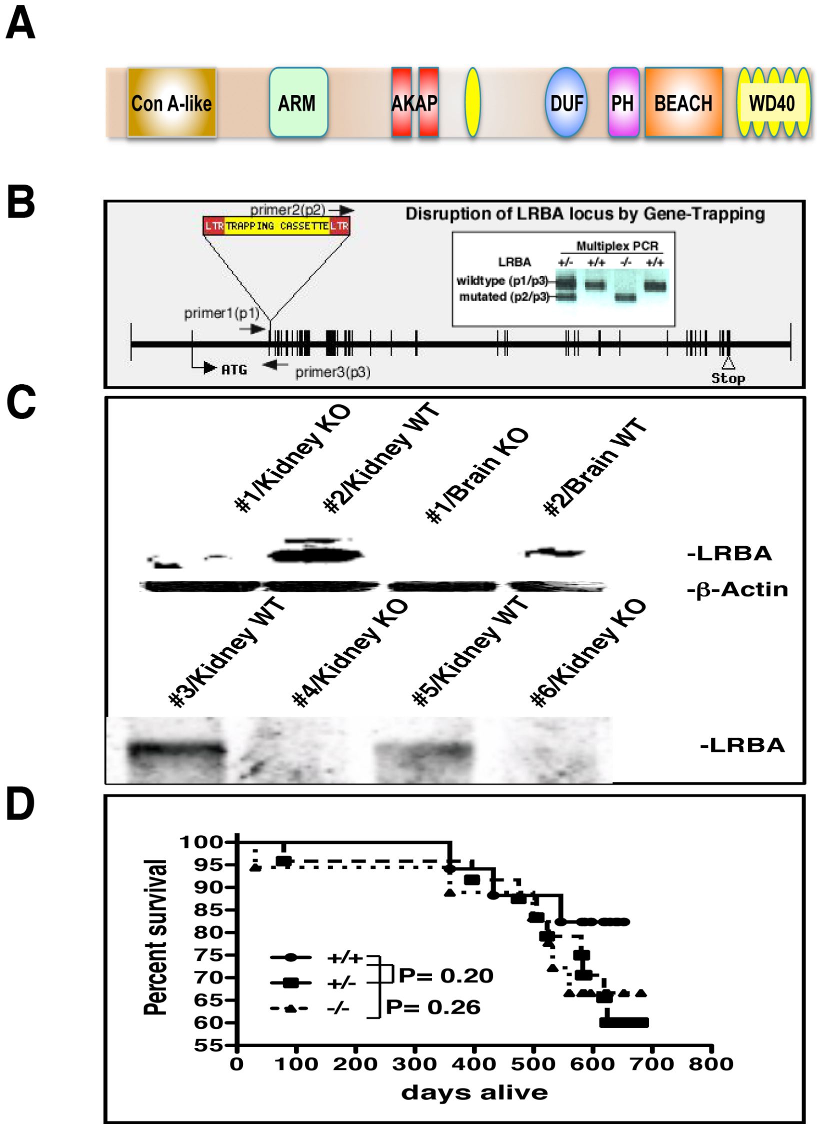

Figure S1. LRBA gene inactivation in mice does not impair longevity.

(A) Schematic representation of the primary structure of LRBA. The Concanavalin A-like lectin (CALL) domain, Armadillo (ARM) domain and the AKAP domain are shown from left to right respectively. The location and approximate size of the DUF (*Domain of Unknown Significance*), PH, BEACH and WD40 domains are shown in the C-terminal portion of LRBA as indicated. *(Schematic has been adapted and modified from (Wang et al., 2001) and (Cullinane et al., 2013) .* **(B)** Diagram showing the site of integration of the gene-trap cassette into the LRBA locus in the intron between exons 2 and 3, the location of the primers used to genotype LRBA$^{-/-}$ mice and an example of PCR genotyping of LRBA null, heterozygous and WT littermates (inset). Primers were designed from the LRBA genomic sequence (p1: GCTCTTGATACTGCCCTGTAGACC and p3: CCCAGCGATGAAAAAGTGGAG) flanking the inserted site and the LTR sequence from the retroviral mutagenesis cassette (p2: AAATGGCTGTACTTAAGCTAGCTTGC). The primer pair (p2/p3) detects the mutated allele. **(C)** Mice homozygous for the LRBA gene-trap integration lack detectable expression of LRBA mRNA. Total RNAs were extracted from kidneys and/or brains from six littermates (#1 to #6) and 20mg of total RNA was loaded into each lane of a Northern blot. Biotin probes of an LRBA fragment spanning nucleotides 5982 to 6450 in the LRBA cDNA and b-Actin were amplified by PCR and labeled with IRDye 800CW Streptavidin and IRDye 680RD Streptavidin (LI-COR Corporate), respectively, followed by a standard Northern hybridization procedure. Northern blot signals were detected by the Odyssey® Infrared Imaging System (LI-COR Corporate). The results confirm the data obtained by PCR genotyping. **(D)** Survival curves for LRBA$^{-/-}$(n=18), WT (n=18) and heterozygous mice(n=24). Mice homozygous for the LRBA inactivating allele (**-/-**) were found to have a normal and healthful lifespan that does not differ significantly from wild-type (**+/+**) and heterozygous (**+/-**) littermates as determined by the Kaplan-Meier log-rank test.

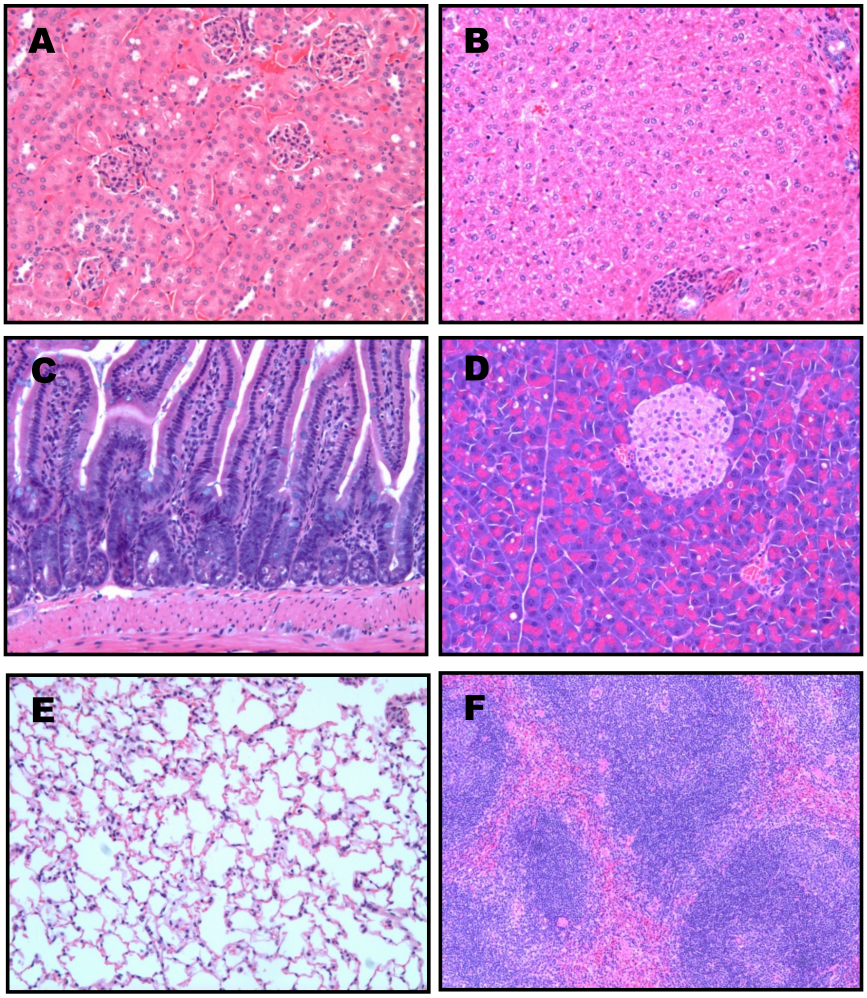

Figure S2. **Histology of representative organs of LRBA-null mice.** Tissues were isolated from LRBA-null mice, fixed in 10% neutral buffered formalin, dehydrated, embedded in paraffin, sectioned and stained with hematoxylin & eosin. Histology of representative organs of LRBA knockout mice including the **(A)** kidney, **(B)** liver, **(C)** small intestine, **(D)** pancreas, **(E)** lung, and **(F)** spleen, each without significant abnormality (H&E, 200x A-E; 100x F) is shown.

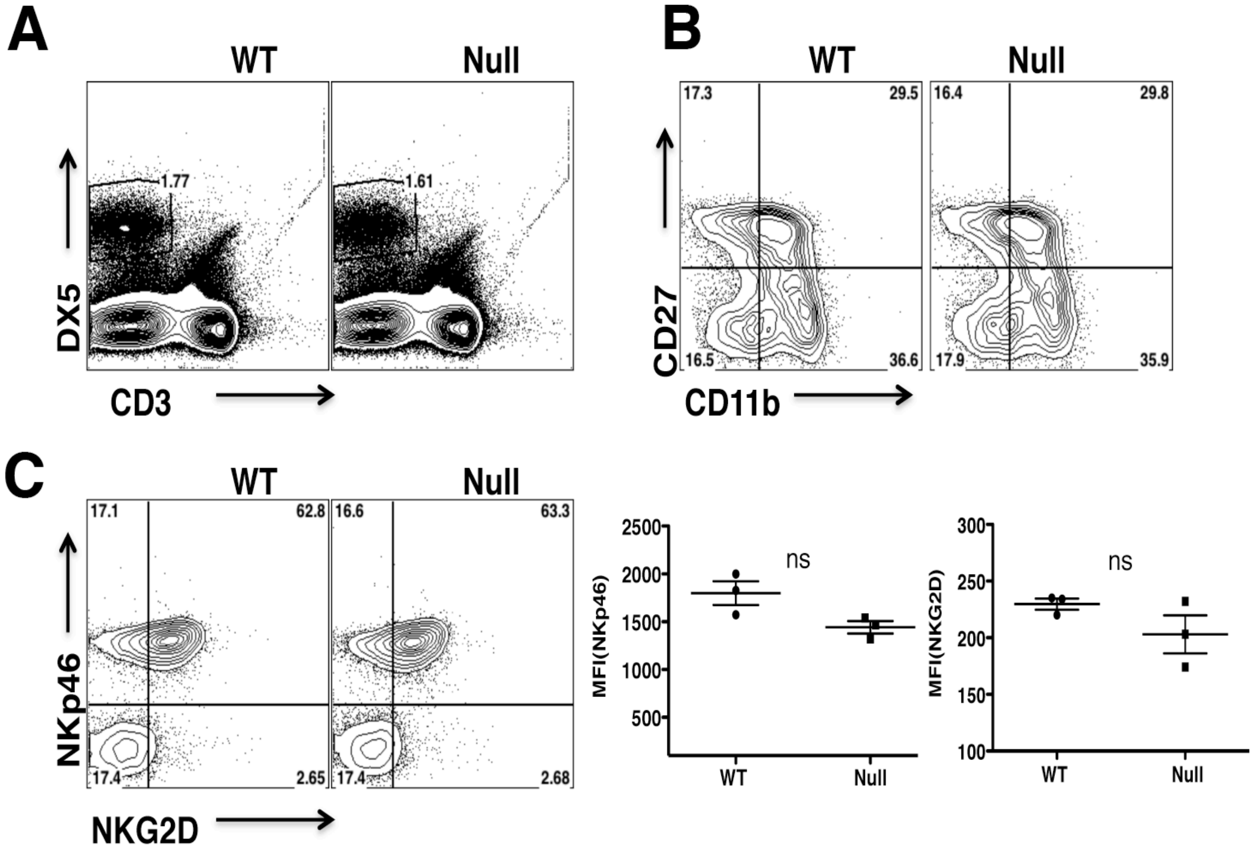

Figure S3. LRBA null mice show normal NK cell numbers, NK maturation and NK activating receptor expression but impaired activating receptor signaling. (A) Representative CD49b(DX5) versus CD3 flow plots from spleens of WT and LRBA-null mice. $DX5^+CD3^-$ gate indicates the frequency NK cells. **(B)** Representative CD11b vs. CD27 contour plots for each genotype as indicated after gating on total NK cells ($DX5^+CD3^-$). **(C)** Representative NKp46 vs. NKG2D contour plots after gating on viable $DX5^+CD3^-$ splenocytes of the indicated genotype and scattered plots showing NKp46 and NKG2D MFI. **(A-C)** Experiment was performed multiple times and representative plots from one experiment with 3 animals per group are shown.

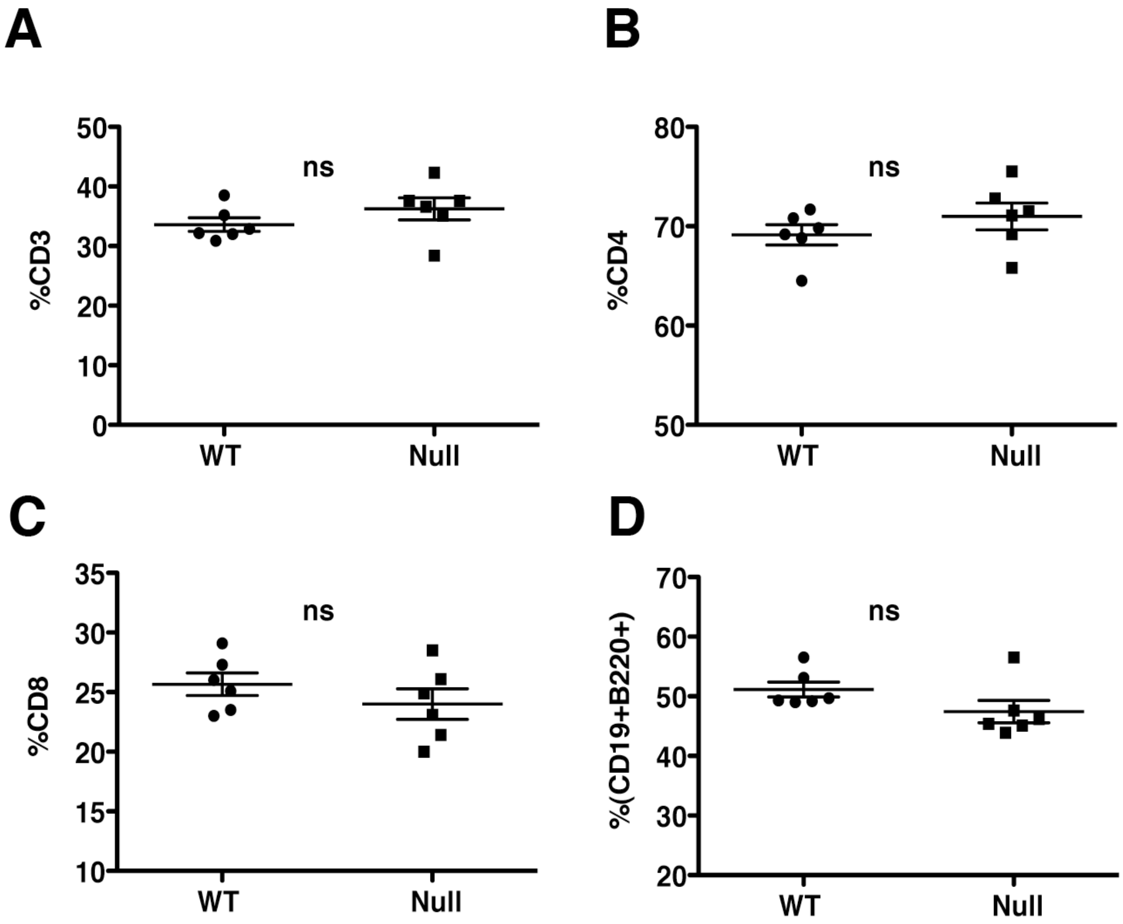

Figure S4. LRBA-null mice have normal T-cell and B cell numbers. Scattered plots showing frequency of CD3+(**A**), CD4+(**B**) CD8+(**C**) T-cells and CD19+B220+(**D**) B-cells from splenocytes of LRBA-null and WT mice. N=6 (pooled data from two experiments is shown.
